# A Novel Interaction between Plant-Beneficial Rhizobacteria and Roots: Colonization Induces Corn Resistance against the Root Herbivore *Diabrotica speciosa*

**DOI:** 10.1371/journal.pone.0113280

**Published:** 2014-11-18

**Authors:** Franciele Santos, Maria Fernanda G. V. Peñaflor, Paul W. Paré, Patrícia A. Sanches, Aline C. Kamiya, Mateus Tonelli, Cristiane Nardi, José Mauricio S. Bento

**Affiliations:** 1 Department of Entomology and Acarology, University of São Paulo, Escola Superior de Agricultura “Luiz de Queiroz”, Piracicaba, SP, Brazil; 2 Departments of Chemistry and Biology, Texas Tech University, Lubbock, TX, USA; 3 Department of Agronomy, Mid-West State University, Guarapuava, PR, Brazil; South China Agricultural University, China

## Abstract

A number of soil-borne microorganisms, such as mycorrhizal fungi and rhizobacteria, establish mutualistic interactions with plants, which can indirectly affect other organisms. Knowledge of the plant-mediated effects of mutualistic microorganisms is limited to aboveground insects, whereas there is little understanding of what role beneficial soil bacteria may play in plant defense against root herbivory. Here, we establish that colonization by the beneficial rhizobacterium *Azospirillum brasilense* affects the host selection and performance of the insect *Diabrotica speciosa*. Root larvae preferentially orient toward the roots of non-inoculated plants versus inoculated roots and gain less weight when feeding on inoculated plants. As inoculation by *A. brasilense* induces higher emissions of (*E*)-β-caryophyllene compared with non-inoculated plants, it is plausible that the non-preference of *D. speciosa* for inoculated plants is related to this sesquiterpene, which is well known to mediate belowground insect-plant interactions. To the best of our knowledge, this is the first study showing that a beneficial rhizobacterium inoculant indirectly alters belowground plant-insect interactions. The role of *A. brasilense* as part of an integrative pest management (IPM) program for the protection of corn against the South American corn rootworm, *D. speciosa*, is considered.

## Introduction

Soil-borne microorganisms, including mycorrhizal fungi and rhizobacteria, can colonize roots and establish mutualistic interactions, inducing a range of plant responses, from growth promotion to pathogen defense [1]. From a plant’s perspective, up to 25% of photosynthesis is allocated toward root exudation, providing an organic-rich nutrient base for the rhizosphere microbiome [2]. Given these changes in plant nutritional quality and defense status, it is expected that mutualistic associations with beneficial microorganisms can impact above- and belowground plant-insect interactions [3].

Knowledge on the plant-mediated effects of mutualistic microorganisms is currently limited to aboveground insects (review by Pineda et al. [4]). Herbivores attacking plants in association with beneficial bacteria can either develop poorly or better depending on both their degree of specialization [5] and the specific plant-beneficial microorganism interaction [3], [6]. For some systems, the association of plants with beneficial microorganisms results in resistance to generalist herbivores, but not always to specialists [7], [8]. However, in other cases, beneficial microorganism-plant mutualism may benefit from herbivore attack [9].

Members of the bacterial genus *Azospirillum* are found in association with plant cereals worldwide [10] and have been used as inoculants for improving crop yields [11], [12]. In Brazil, *Azospirillum brasilense* Tarrand, Krieg and Döbereiner (Rhodospirillaceae) is the predominantly applied plant growth-promoting rhizobacterium (PGPR) in corn and wheat crops [13]. Despite the well-established beneficial effect of *Azospirillum* on crop plant growth and resistance to pathogens, the impact of inoculant application on herbivore performance and behavior is not well understood.

The South American corn rootworm, *Diabrotica speciosa* (Germar) (Coleoptera: Chrysomelidae), is a polyphagous herbivore, particularly during the adult stage, when this species causes severe damage to corn (*Zea mays* L.), beans (*Phaseolus* spp.) and soybeans (*Glycine max* (L.) Merrill) [14]. The larval stage lives in the soil and is usually found in corn roots, where its development is optimal compared with in other crop plants [15].

Considering the substantial damage inflicted by *D. speciosa* larvae to corn roots in Brazil and the wide use of *A. brasilense* as an inoculant, the present study investigated the plant-mediated effects of *A. brasilense* in corn on the host selection and performance of *D. speciosa* larvae. As host choice by rhizophagous insects is guided by root volatiles in particular [16], we also assessed whether root volatile emissions are altered in response to colonization by *A. brasilense*. To the best of our knowledge, this is the first study showing that a beneficial rhizobacterium inoculant indirectly alters belowground plant-insect interactions.

## Materials and Methods

### Plant Growth Conditions and Inoculation

Corn seeds *(Z. mays*, variety Delprim; Delley Semences et Plants SA, Delley, Switzerland) were inoculated with *Azospirillum brasilense* (strains AbV5 and AbV6) via mixture with Nitro 1000 (Cascavel, PR, Brazil) at a concentration of 1×10^7^ colony formation units (CFU)/mL. Although *A. brasilense* is commercially referred to as a plant-growth-promoting rhizobacterium (PGPR), we did not detect any plant growth promotion, at least in terms of root and shoot biomass, within 10 days after inoculation (Figures S1 and S2). Therefore, we have adopted the term plant-beneficial rhizobacteria throughout this work. Control corn seeds were prepared in the same way as inoculated seeds but using distilled water in place of the inoculum. Seeds were sown in Basiplant potting soil (250 cm^3^) with no additional fertilization and were grown in an insect-free greenhouse from July to October 2013 (winter-spring) under natural light (Piracicaba, SP, Brazil).

### Insect Rearing

Adults of the South American corn rootworm, *D. speciosa*, were collected in the field (from cucumber crops in Piracicaba, SP, Brazil, 22°43′14″ to 22°42′ 01″ S and 47°38′46″ to 47°36′49″ W, where the insect is an agricultural pest and no specific permission is required) and multiplied under laboratory conditions (25°C, 60% RH, 12 L:12 D) for three to five generations. Briefly, adults were fed on common beans (*Phaseolus vulgaris* L.) and larvae on corn seedlings. Eggs were collected every other day from black-dyed gauze that served as a substrate for oviposition and then transferred to Petri dishes with wet filter paper until hatching. Newly hatching larvae were immediately transferred to corn seedlings germinated in vermiculite. After approximately 30 days, adults emerged. For details on the method used for *D. speciosa* laboratory rearing, see reference [17]. The field collections involved only *D. speciosa* adults and therefore did not involve any endangered or protected species.

### Olfactometer Assays

Host selection by *D. speciosa* larvae were evaluated in a glass six-arm olfactometer consisting of a central chamber (8 cm length, 10 cm diameter) with six arms connected to side chambers (5 cm length, 3 cm diameter) [18]. Three days before the bioassay, 7- to 8-day-old corn seedlings were transferred to the olfactometry side chambers, and the remaining space was filled with a mixture of sterile sand and rocks, in a 2∶1 ratio, moistened with 10% water (dry sand:water; g/g). Each side chamber containing wet soil and seedlings was weighed before being transferred to the greenhouse. To maintain the moisture content at approximately 10% during the three days in the greenhouse, we weighed the chamber set daily and added the appropriate volume of water to return it to its initial weight. The treatment chambers were alternated with controls, and a total of 30 second-third instar *D. speciosa* larvae were released in the central chamber, where they could freely choose among the 6 chambers over 24 hours. Thereafter, the olfactometer was disassembled, and the number of larvae in each side chamber was registered. When a larva did not leave the central chamber, it was recorded as a non-choice. Each assay replicate employed fresh plants and insect sets.

### Larval Performance

Newly hatched *D. speciosa* larvae were allowed to feed on 5-day-old inoculated or non-inoculated corn seedlings grown in pots containing sterile vermiculite (25±1°C, 60% RH, 12 L:12 D). Each pot contained 30 corn seedlings, with one larva/seedling. The larvae were recovered ten days after feeding and weighed individually.

### Volatile Collection and Analysis

The shoots and roots of inoculated and non-inoculated plants were harvested and weighed, and the roots were flash frozen in N_2(l)_ and stored for volatile analysis. To collect volatiles, frozen roots were ground into a powder in liquid nitrogen, and a 30 mg aliquot was transferred to a 4 mL screw cap septum vial. According to Rasmann et al. [18], an SPME syringe carrying 100 µm polydimethylsiloxane (PDMS) (Supelco, Bellefonte, PA, USA) was used to puncture the septum, and the tissue was exposed for 60 minutes at 25°C. The SPME fiber was then immediately subjected to GC injection into an HP5-MS capillary column (JeW Scientific, Folsom, CA; 30 m×0.25 mm×0.25 µm) using Helium as the carrier gas. The SPME fiber was held in the injector (250°C) for 5 min to completely elute volatiles from the fiber. The column temperature was maintained at 40°C for five min, then increased to 150°C (5°C/min) for one min, then raised again (5°C/min) until reaching the final temperature of 250°C. Compounds were MS identified (Varian 4000) based on a comparison of the obtained mass spectra and retention times with those of authentic standards and the Kovats Index (KI) using *n*-alkane (C_7_–C_30_) standards [19] (Table 1).

**Table 1:** Identification of compounds in the root volatile profile.

Identified compound	Rt (min)	KI	%	
			Inoculated	Non-inoculated
decanal	20.351	1206	6.68	22.66
unknown hydrocarbon I	23.968	1336	10.54	11.38
unknown hydrocarbon II	24.240	1346	6.84	6.56
α-copaene	25.136	1380	4.69	4.92
1-tetradecene	25.450	1393	10.96	17.74
(*E*)-β-caryophyllene	26.289	1424	50.85	22.15
geranyl acetone	27.026	1454	9.44	14.60

Retention time (Rt), Kovats Index (KI), peak area (%) and identification of compounds emitted by inoculated and non-inoculated corn roots through combined GC-MS analysis.

### Scanning Electron Microscopy

The roots of inoculated and non-inoculated corn were cut, rinsed in tap water and fixed in 3% glutaraldehyde in 50 mM cacodylate buffer (pH 7.0). Tissue dehydration was performed in a graded series ending in absolute acetone, followed by critical point drying. Then, the samples were mounted on stubs and subjected to gold sputtering (SDC-050 Sputter Coater, BAL-TEC). Finally, the samples were analyzed on a Zeiss LEO 435 VP scanning electron microscope.

### Statistical Analysis

Kolmogorov–Smirnov and Levene’s tests were carried out to determine the normality and homogeneity of the data. Data from the olfactometer choice assays and the larval weights obtained in the performance experiments were analyzed using either general log-linear model (glm) or general log-linear mixed model (glmm) (*P*<0.05). The relative amounts of volatile compounds released by inoculated and control corn roots were Log_10_(x+1) transformed and analyzed through *One-Way* ANOVA followed by Tukey’s HSD (*P*<0.05). The statistical tests were performed using the software package R (www.R-project.org) version 2.8.1 and Minitab Release 14.

## Results

**Figure 1:**
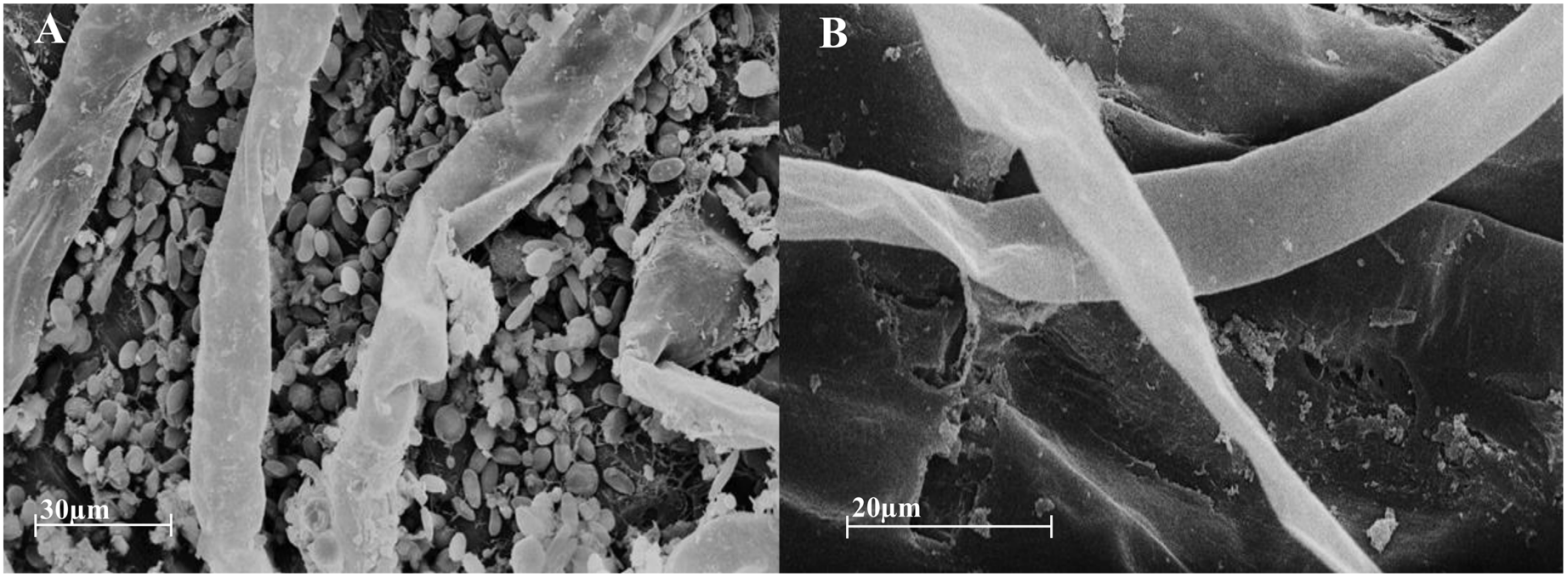
Scanning electron microscopy images of inoculated and non-inoculated corn roots. Scanning electron microscopy images showing the colonization of corn roots by the plant-beneficial rhizobacterium *Azospirillum brasilense*. (A) Inoculated corn roots and (B) non-inoculated corn roots.

**Figure 2:**
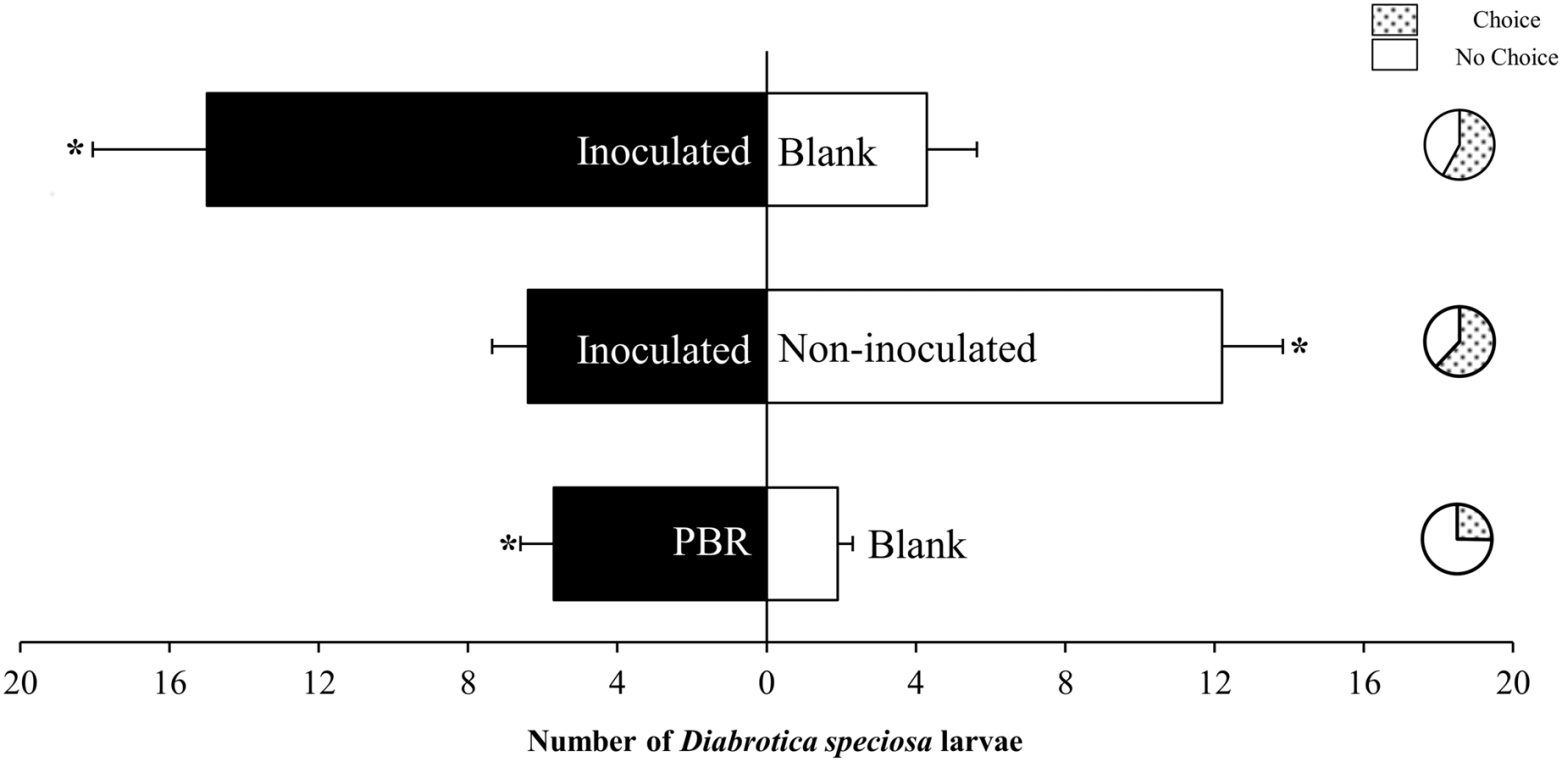
Effect of *Azospirillum brasilense* on *Diabrotica speciosa* larval host choice. *Diabrotica speciosa* larval choice between inoculated plants and the blank treatment (non-inoculated soil), inoculated and non-inoculated corn, and the plant-beneficial rhizobacterium (PBR) inoculant and the blank. Bars represent the mean number of larvae ± SE. Pie charts on the right represent non-responsive (no choice) and responsive (choice) larvae. Asterisks indicate a significant difference between treatments according to a quasi-Poisson glm (n = 10, *P*<0.05).

**Figure 3:**
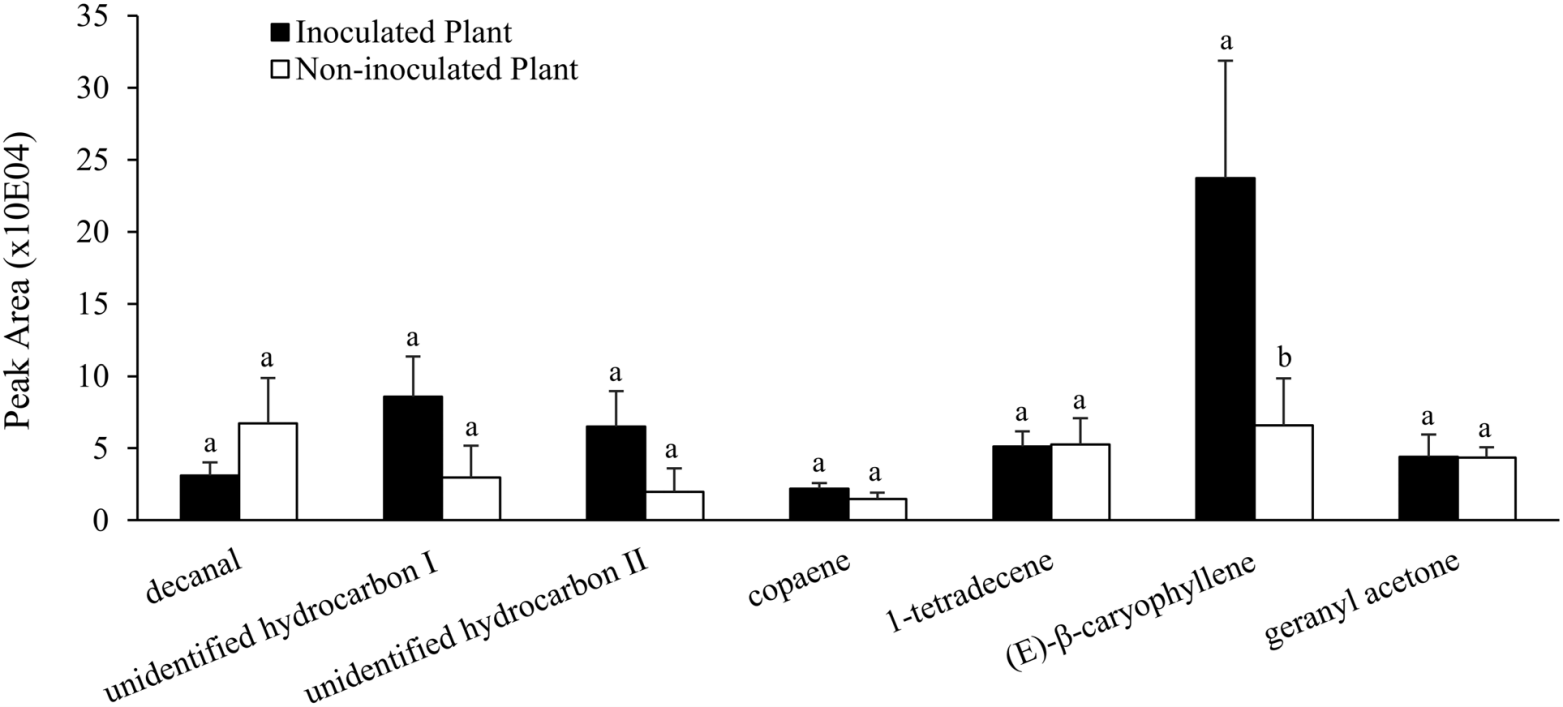
Root volatile profile induced by *Azospirillum brasilense* colonization. Emissions of volatile compounds from inoculated and non-inoculated corn roots. Bars represent the mean ± SE. Different letters indicate a significant difference between treatments according to *One-Way* ANOVA followed by Tukey’s HSD test (n = 4, *P*<0.05).

Scanning electron microscopy revealed that the roots of inoculated plants were successfully colonized by *A. brasilense* (Figure 1). To determine whether corn roots inoculated with *A. brasilense* influence insect behavior, a six-arm olfactometer was employed, in which South American corn rootworm larvae were allowed to move through soil to one of two stimulus choices. Regarding the choice of plants that were either inoculated or not inoculated with *A. brasilense,* the beetle larvae preferred non-inoculated plants (Figure 2, n = 10; glm, *F_1,18_* = 10.13, *P* = 0.005), which emitted 72% less (*E*)-β-caryophyllene (Figure 3). To confirm that the outcome of the interaction was not a result of odors derived from the inoculant itself, larvae were presented with soil either inoculated with *A. brasilense* or not, but few larvae left the central chamber (75% of larvae were non-responsive), and the responsive larvae preferentially chose arms with the inoculant (Figure 2, n = 10; glm, *F_1,18_* = 17.46, *P*<0.001). When larvae were allowed to choose between non-inoculated soil (blank) and inoculated plants, the larvae preferentially oriented toward the inoculated plants (Figure 2, n = 3; glm, *F_1,4_* = 12.06, *P* = 0.025). Therefore, in the absence of a suitable host, the larvae oriented toward non-preferred hosts to survive. No chamber bias was observed, with an equal larval distribution between soil without plants and soil with non-inoculated plants (Figure S3).

To determine the role of the plant-beneficial rhizobacterium interaction in belowground plant defenses against insect pests, the root volatile profile was chemically characterized and quantified in *A. brasilense* inoculated roots (Table 1). The sesquiterpene (*E*)-β-caryophyllene was detected at a 3.6-fold higher level in inoculated plants than in the non-inoculated control via *in vitro* SPME analysis (Figure 3 and Table 1, Tukey’s HSD, *One-Way* ANOVA, *F_1,6_* = 7.96, *P* = 0.030). Other volatile metabolites detected in the corn roots included decanal, copaene, 1-tetradecene and geranyl acetone as well as two unidentified hydrocarbons that were not induced by the plant-beneficial rhizobacteria (Figure 3 and Table 1).

**Figure 4:**
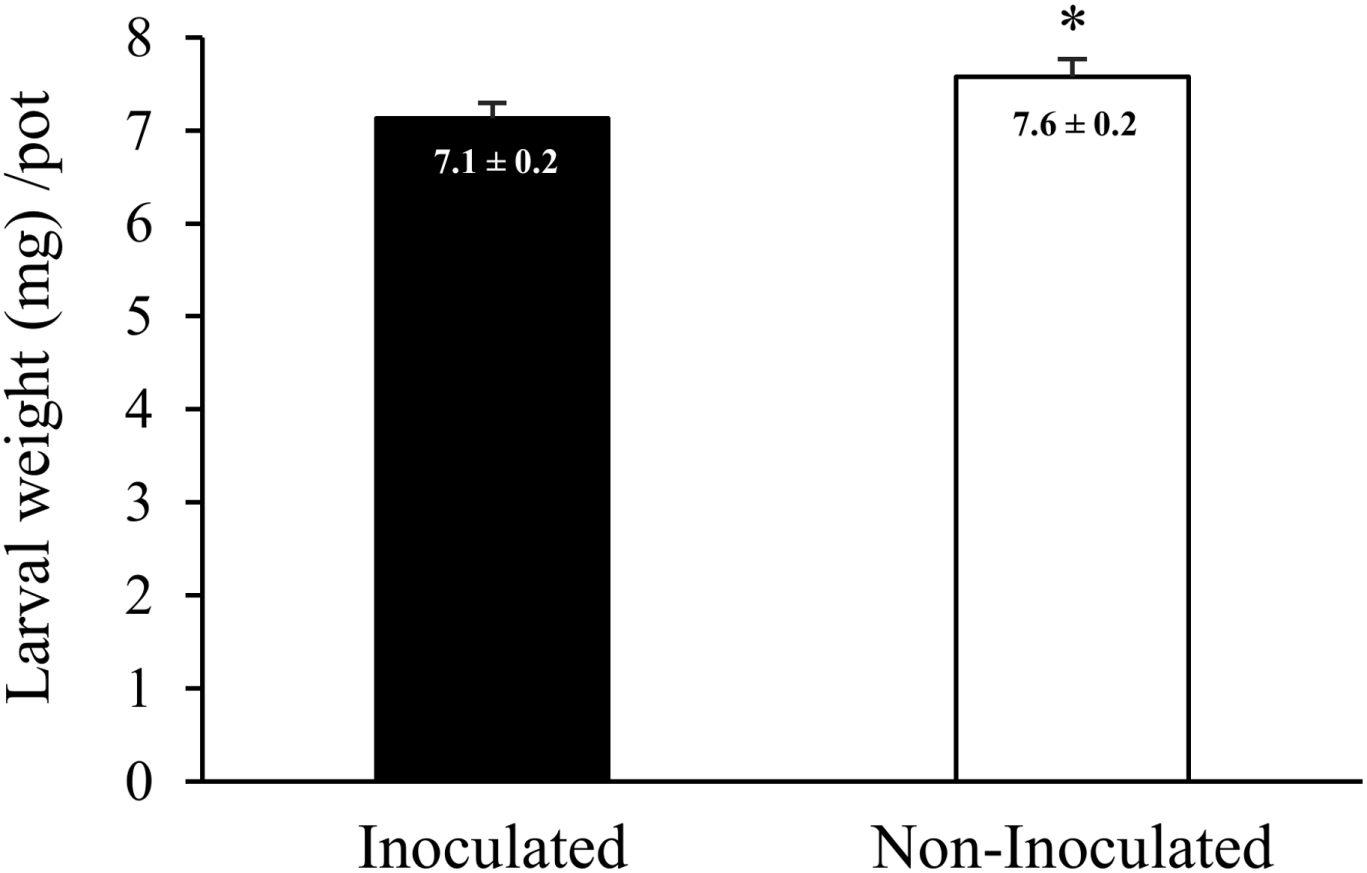
Effect of *Azospirillum brasilense* on *Diabrotica speciosa* performance. *Diabrotica speciosa* larval performance when fed on inoculated and non-inoculated corn plants. Bars represent the mean larval weight ± SE. Asterisks indicate a significant difference between treatments according to according to a glmm (n = 10, *P*<0.05).

In addition to testing larval host selection for inoculated and non-inoculated plants, larval performance was assayed by monitoring larval weight in relation to diet. The *D. speciosa* larvae were heavier when they fed on non-inoculated plants than on inoculated plants (Figure 4, n = 10; glmm *F_1,432_* = 5.05 *P* = 0.025).

## Discussion

Root colonization by rhizobacteria induces plant resistance against pathogens in numerous agricultural systems [20] as well as plant protection against herbivory in a number of examined crops [3], [21], [22], [23]. Previous studies on rhizobacterium- and mycorrhizal-inducible plant volatiles have primarily focused on aboveground plant-herbivore interactions [24], [25].

The present study shows that colonization by the plant-beneficial rhizobacterium *A. brasilense* alters host choice by *D. speciosa* larvae, which preferentially oriented toward the roots of non-inoculated versus inoculated plants (Figure 2). As odors from the inoculant itself were attractive to the larvae (Figure 2), it is clear that the effect of plant inoculation on *D. speciosa* behavior was associated with the beneficial rhizobacterium-plant interaction. Odors produced by plant-beneficial soil bacteria are known to induce plant defenses [26] and may play a role in interactions with higher trophic levels [27]. However, as the inoculant used here contains components responsible for maintaining bacterial colony survival, it was not possible to infer that bacterial odors act as attractants for *D. speciosa* larvae.

In the present study, we established that root colonization by the plant-beneficial rhizobacterium *A. brasilense* specifically augments (*E*)-β-caryophyllene emissions in corn roots. The larvae of *D. v. virgifera* can exploit (*E*)-β-caryophyllene concentrations as a signal to select corn plants infested by a suitable number of conspecifics [28]. Therefore, larval behavior ranges from attraction to repellence by (*E*)-β-caryophyllene emissions depending on host nutritional quality and defense status. Given that the performance of *D. speciosa* is poorer when feeding on plants inoculated with plant-beneficial rhizobacteria versus non-inoculated plants (Figure 4), elevated (*E*)-β-caryophyllene root emissions may provide a chemical signal for larvae to avoid unsuitable hosts.

Indeed, entomopathogenic nematodes have been shown to use elevated levels of (*E*)-β-caryophyllene released from *D. virgifera*-damaged roots as a chemical cue in host location [18]. In addition to (*E*)-β-caryophyllene mediating subterranean tritrophic interactions between plants, herbivores and natural enemies of herbivores, this biologically active signal also functions in plant pathogen defense to inhibit microbial and fungal growth and may be rhizobacterium induced during the activation of induced systemic induction [29], [30].

Although *A. brasilense* inoculated corn has been reported to exhibit an enhanced biomass [31], we did not observe growth promotion in inoculated ten-day-old seedlings (Figures S1 and S2), as this effect may occur in late developmental stages of corn [8]. Therefore, plant biomass alone did not directly impact larval performance or preference. It is more likely that the low larval weight associated with feeding on inoculated plants is due to induced plant defenses, though metabolic profiling has yet to be performed. Nonetheless, we cannot discard the possibility that the root-colonizing rhizobacterium *A. brasilense* displays entomopathogenicity [32].

In general, our data show that the beneficial rhizobacterium *A. brasilense* confers protection to corn plants against attack by *D. speciosa* larvae. It has been established that treatment of cucumber with plant-beneficial rhizobacteria results in greater control of populations of *Diabrotica* sp. compared with non-treated plots and even chemical control [21]. In addition, elevated emissions of (*E*)-β-caryophyllene from undamaged inoculated corn may recruit entomopathogenic nematodes, *contributing to Diabrotica* control. The potential for employing plant-beneficial rhizobacteria such as *A. brasilense* as a component of an integrative pest management (IPM) program for protection of corn against *D. speciosa* clearly warrants further investigation.

## Supporting Information

Figure S1 **Shoot biomass of inoculated and non-inoculated corn plants.** Dry weight of the shoots of inoculated and non-inoculated corn plants (mean ± SE). No significant difference was detected according to Student’s *t-test* (n = 6).
(TIF)

Figure S2 **Root biomass of inoculated and non-inoculated corn plants.** Fresh weight of the roots of inoculated and non-inoculated corn plants (mean ± SE). No significant difference was detected according to Student’s *t-test* (n = 6).
(TIF)

Figure S3 ***Diabrotica speciosa*** **larval choice in control assays in a six-arm olfactometer.** *Diabrotica speciosa* larval choice in control assays using non-inoculated plants *vs*. non inoculated plants (control) and blank *vs*. blank (no stimulus). Bars represent the mean number of larvae ± SE. The pie charts on the right represent non-responsive (no choice) and responsive (choice) larvae. No significant difference was detected according to a quasi-Poisson glm (n = 5).
(TIF)

Figure S4 **Density of the distribution of larval weight data.** Density of the distribution of raw data on the weight of *Diabrotica speciosa* when fed on non-inoculated (pink area - control) and inoculated (light green area - inoc) corn. A total of 444 data points: 227 for larval weight on non-inoculated corn and 217 on inoculated corn.
(JPG)

File S1 **Data set of** ***Diabrotica speciosa*** **host choice, larval performance, corn root volatile emissions, and root and shoot biomass.**
(XLSX)
