# Supplementary figures and images for: A Novel Interaction between Plant-Beneficial Rhizobacteria and Roots: Colonization Induces Corn Resistance against the Root Herbivore Diabrotica speciosa
Source: PLoS One. 2014 Nov 18;9(11):e113280. doi: 10.1371/journal.pone.0113280 (PMC4236168; doi:10.1371/journal.pone.0113280)

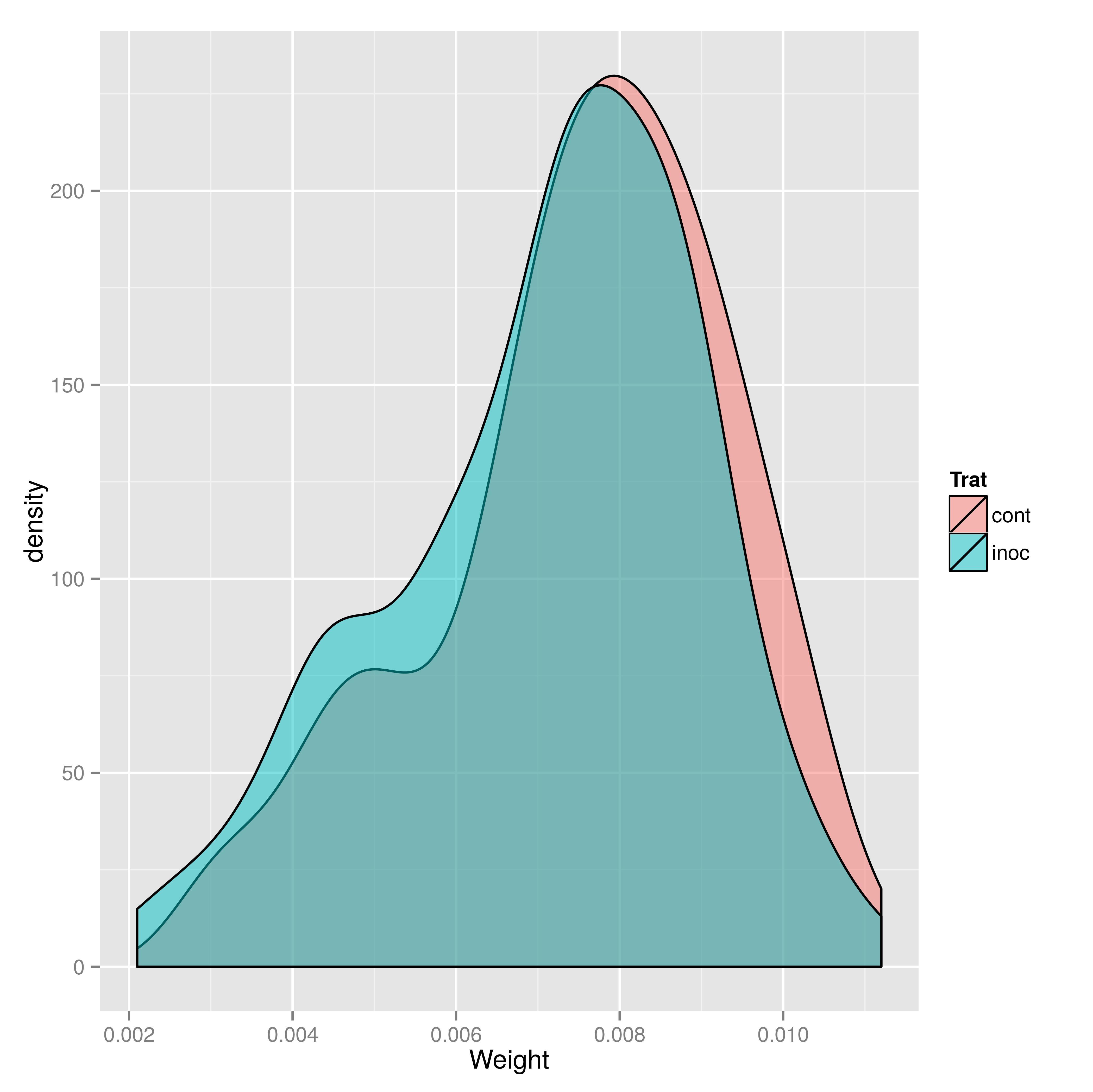

Supplement: Figure S4 — Density of the distribution of larval weight data. Density of the distribution of raw data on the weight of Diabrotica speciosa when fed on non-inoculated (pink area - control) and inoculated (light green area - inoc) corn. A total of 444 data points: 227 for larval weight on non-inoculated corn and 217 on inoculated corn. (JPG) [file pone.0113280.s004.jpg]
